# Differential Function of Lip Residues in the Mechanism and Biology of an Anthrax Hemophore

**DOI:** 10.1371/journal.ppat.1002559

**Published:** 2012-03-08

**Authors:** MarCia T. Ekworomadu, Catherine B. Poor, Cedric P. Owens, Miriam A. Balderas, Marian Fabian, John S. Olson, Frank Murphy, Erol Balkabasi, Erin S. Honsa, Chuan He, Celia W. Goulding, Anthony W. Maresso

**Affiliations:** 1 Department of Molecular Virology and Microbiology, Baylor College of Medicine, Houston, Texas, United States of America; 2 Department of Chemistry, University of Chicago, Chicago, Illinois, United States of America; 3 Departments of Molecular Biology and Biochemistry, University of California-Irvine, Irvine, California, United States of America; 4 Department of Biochemistry and Cell Biology, Rice University, Houston, Texas, United States of America; 5 Northeastern Collaborative Access Team, Argonne National Laboratory, Argonne, Illinois, United States of America; Tufts University School of Medicine, United States of America

## Abstract

To replicate in mammalian hosts, bacterial pathogens must acquire iron. The majority of iron is coordinated to the protoporphyrin ring of heme, which is further bound to hemoglobin. Pathogenic bacteria utilize secreted hemophores to acquire heme from heme sources such as hemoglobin. *Bacillus anthracis*, the causative agent of anthrax disease, secretes two hemophores, IsdX1 and IsdX2, to acquire heme from host hemoglobin and enhance bacterial replication in iron-starved environments. Both proteins contain NEAr-iron Transporter (NEAT) domains, a conserved protein module that functions in heme acquisition in Gram-positive pathogens. Here, we report the structure of IsdX1, the first of a Gram-positive hemophore, with and without bound heme. Overall, IsdX1 forms an immunoglobin-like fold that contains, similar to other NEAT proteins, a 3_10_-helix near the heme-binding site. Because the mechanistic function of this helix in NEAT proteins is not yet defined, we focused on the contribution of this region to hemophore and NEAT protein activity, both biochemically and biologically in cultured cells. Site-directed mutagenesis of amino acids in and adjacent to the helix identified residues important for heme and hemoglobin association, with some mutations affecting both properties and other mutations affecting only heme stabilization. IsdX1 with mutations that reduced the ability to associate with hemoglobin and bind heme failed to restore the growth of a hemophore-deficient strain of *B. anthracis* on hemoglobin as the sole iron source. These data indicate that not only is the 3_10_-helix important for NEAT protein biology, but also that the processes of hemoglobin and heme binding can be both separate as well as coupled, the latter function being necessary for maximal heme-scavenging activity. These studies enhance our understanding of NEAT domain and hemophore function and set the stage for structure-based inhibitor design to block NEAT domain interaction with upstream ligands.

## Author Summary

Pathogenic bacteria need to acquire host iron to replicate during infection. Approximately 80% of mammalian iron is associated with a small molecule termed heme, most of which is bound to circulating hemoglobin and involved in O_2_ transport in red cells. Bacteria secrete proteins, termed hemophores, to acquire the heme from hemoglobin, a process thought to accelerate delivery of the heme to the bacterial surface for iron import into the cell. The mechanisms by which hemophores extract host heme from hemoglobin are not known. Here, we report that the IsdX1 hemophore from *B. anthracis*, the causative agent of anthrax disease, uses a conserved structural feature to link hemoglobin association with heme binding and extraction, thereby facilitating bacterial growth in low-iron environments. Such “molecular coupling” suggests that specific inhibition of the hemophore-hemoglobin interaction for this class of proteins may serve as a starting point for new anti-infective therapeutics aimed at short-circuiting iron uptake networks in bacterial pathogens.

## Introduction

An important determinant in the outcome of a bacterial infection is how well the invading pathogen can acquire host iron. Hosts with high levels of free iron are more susceptible to infection, and deletion of iron acquisition systems in a wide range of bacterial species generally attenuates virulence [1]. The low free iron concentration in host tissues (10^−18–24^ M) likely acts as a barrier to efficient bacterial replication [2]. However, pathogenic bacteria have evolved at least two distinct uptake systems to attain iron. One such mechanism is to secrete siderophores, small molecules that chelate ferric iron with very high affinity [3]. The iron-bound siderophore binds to the bacterial surface and specific ferric iron receptors next deliver the iron or iron-siderophore complex into the cell [4]. The genetic deletion of biosynthetic systems that make siderophores, or the surface receptors that recognize siderophores, decreases the virulence of several pathogens, including the Gram-positive bacteria *B. anthracis* [5] and *S. aureus* [6].

The second system bacteria employ to attain host iron targets iron-protoporphyrin IX, or heme. Although heme constitutes up to 80% of the bodily iron reserves, free heme is rare. Most heme is tightly bound to hemoproteins such as hemoglobin. Hemoglobin's important role as an oxygen carrier protein means it is in high abundance and thus a target for bacterial iron uptake [7]–[9]. Bacterial proteins that acquire heme from hemoglobin are called hemophores [10]–[13]. Hemophores are generally secreted into the external milieu where they extract heme, via an undefined mechanism, from heme sources such as hemoglobin [14]. The heme-bound (holo) hemophore then delivers its bound iron-porphyrin to a cognate receptor on the bacteria surface, which leads to heme import into the bacterial cell [15], [16]. Heme from hemoglobin can also be attained at the bacterial surface through similar mechanisms involving receptors on the cell wall (Gram positive) or outer membrane (Gram negative). Heme import by bacterial pathogens is important for the establishment or maintenance of infections caused by *Bordetella* [17], *Haemophilus* [18], *Brucella* [19], *Vibrio* [20], *Streptococcus* [21], and *Staphylococcus* [22] species. Further, more recent studies suggest heme is a major determinant in the promotion and severity of bacterial sepsis [23]. Collectively, these studies highlight the important role of heme acquisition during infection of mammalian hosts and support the contention that the inhibition of iron uptake systems is a promising direction for the development of new therapeutics. However, despite this knowledge, no clinically-used antibiotics have been made that directly target bacterial iron import. An understanding of the molecular mechanism by which heme acquisition systems extract, bind, and transfer heme into bacterial cells would be an important first step in creating new drugs to treat deadly infections.

The first discovered hemophore was HasA from the Gram-negative pathogen *S. marcescens* [10]. HasA is a small ∼15 kDa protein that seems to passively acquire heme from hemoglobin by virtue of its high affinity for the iron-porphyrin [24]. HasA delivers its bound heme to HasR, an outer membrane surface receptor that delivers the heme into the cell [25]. Whereas the discovery of HasA set the precedence for the study of hemophores, recent studies indicate that Gram-positive bacteria also utilize hemophores. *B. anthracis*, a Gram-positive pathogen that is a potential weapon for bioterrorism, secretes two hemophores (IsdX1 and IsdX2), that acquire heme from hemoglobin and promote bacterial growth in low-iron environments [26]–[28]. IsdX1 transfers its bound heme to IsdC, a surface protein covalently attached to the peptidoglycan of the cell wall [29], [30]. The heme acquisition functions of IsdX1, IsdX2, and IsdC are dependent on the activity of their NEAT (near-iron transporter) domain [26], [29]. This approximately 125 amino acid domain is conserved in several Gram-positive bacteria, and the collective action of several NEATs on the bacterial surface is hypothesized to lead to transfer of heme through the cell wall [27], [31]–[33]. The deletion of genes encoding for NEAT proteins leads to reduction in virulence in both *B. anthracis* and *S. aureus* [34]–[36]. Further, vaccination of mice with recombinant proteins containing NEAT domains induces adaptive immunity to staphylococcal infection. [37]–[39]. However, the development of a safe vaccine or a small molecule inhibitor of NEATs will require a detailed understanding of how NEAT proteins perform their various heme transport functions, including a structural appreciation of the residues required for activity. At the heart of this activity is an understanding of how bacterial hemophores like IsdX1 mediate the specific extraction of heme from host hemoglobin, and whether the ability to take up heme is coupled to the process of heme removal from the globin donor. The NEAT domain of IsdX1 is the only known NEAT domain to bind both heme and hemoglobin, as well as acquire heme from hemoglobin, thus making IsdX1 a useful model protein to study the relationship of these processes to one another. To better understand the molecular basis of hemophore activity and provide mechanistic insights into NEAT protein function, we solved the structures of heme free and heme bound IsdX1 and used these structures to understand the functional roles of residues in a small 3_10_-helix in the vicinity of the heme binding site.

## Results

### The apo and holo structure of IsdX1 highlights residues involved in hemophore function

**Figure 1:**
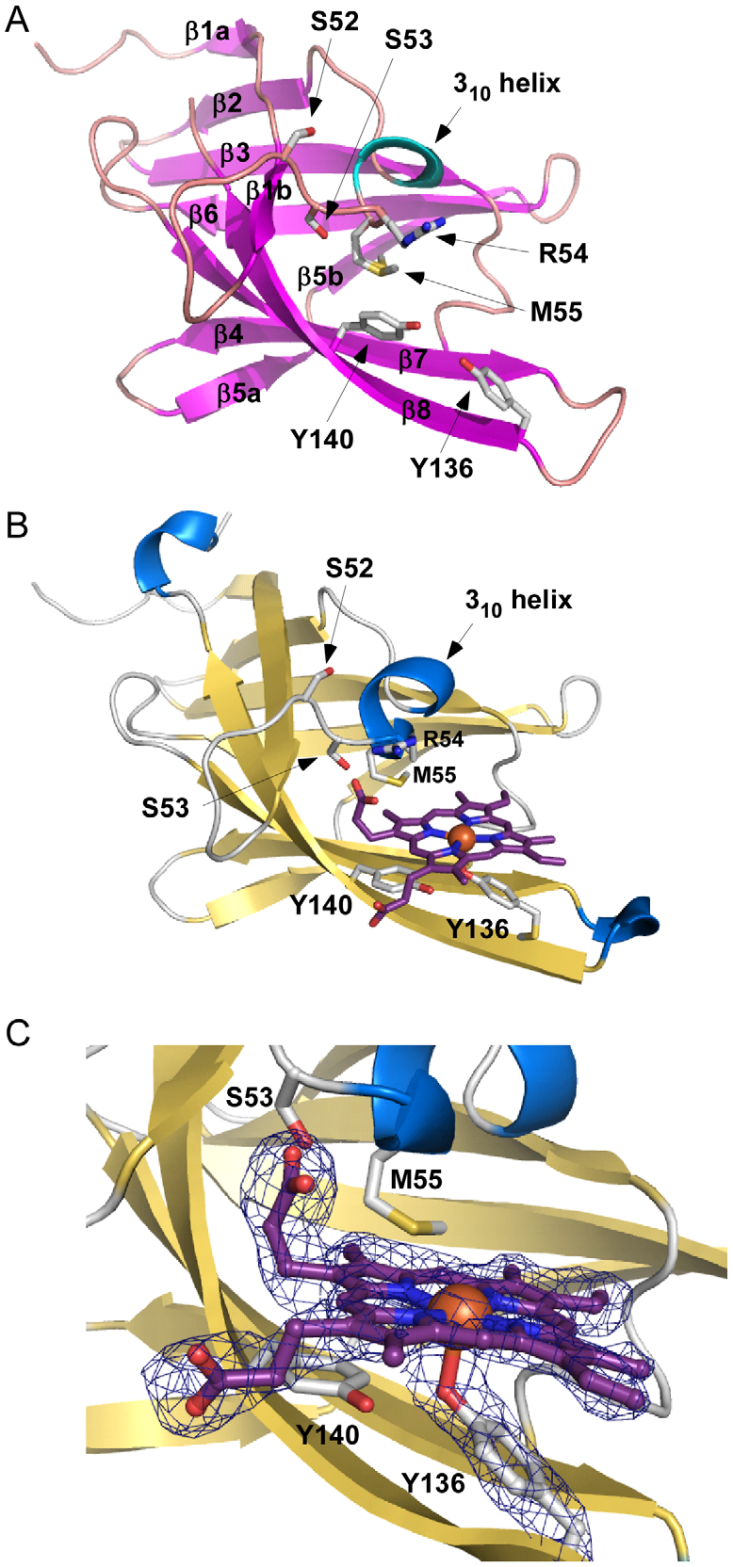
Apo and holo IsdX1 crystal structures. (A) Ribbon representation of apo-IsdX1 where β-strands and helices are colored pink and cyan, respectively. Important heme-binding residues are represented in stick model with carbon, sulfur, nitrogen, and oxygen atoms colored white, yellow, blue and red, respectively. (B) Cartoon representation of holo-IsdX1 where β-strands and helices are colored gold and blue. Heme (purple) is depicted in stick model with carbon (purple), nitrogen (blue), oxygen (red) and Fe (orange sphere). (C) Heme-binding pocket with *F_o_−F_c_* omit map contoured at 3.0 σ (dark blue mesh). Hydrogen bonds are indicated by black dashed lines.

To gain an appreciation of the residues in IsdX1 necessary for the hemophore activity of this protein, we solved the crystal structure of apo-IsdX1 to 1.8 Å resolution (Table 1). Overall, the backbone structure of IsdX1 is similar to the solved structures of NEAT domains from *S. aureus*, albeit with a unique surface charge distribution close to the heme binding pocket (Figure S1.) [40]–[44]. The structure consists of an immunoglobulin-like fold with eight β-strands arranged in two antiparallel β-sheets of a β-sandwich (Figure 1A).

**Table 1:** Crystallography statistics.

Data Collection, Phasing, and Refinement Statistics			
	Apo	SeMet	Holo
**Data collection/parameters**			
Space group	P1	P1	P4_3_
Wavelength (Å)	0.97929	0.97940	1.0
Cell dimensions			
a, b, c (Å)	36.6, 43.7, 47.4	37.0, 43.9, 47.7	64.98×64.98×74.2
α, β, γ (°)	99.24, 96.53, 107.35	99.14, 97.44, 106.37	
Resolution (Å)	46.1-1.80	34.5-2.11	50.0-2.15
Completeness (%)^a^	97.2 (94.4)	97.0 (96.3)	99.1 (100.0)
Unique reflections	24540 (2336)	24211 (250118)	16738 (251543)
R_merge_ (%)^b^	4.5 (35.9)	7.4 (55.1)	4.4 (33.1)
I/σ^a^	18.1 (1.7)	10.9 (2.3)	43.79 (7.23)
Redundancy^a^	2.0 (1.9)	3.9 (3.6)	15.0 (15.1)
**SAD Phasing**			
Resolution (Å)		34.5-2.11	
Selenium sites (#)		2	
Figure of merit (before/after density modification)		0.32/0.60	
**Refinement**			
Resolution (Å)	25.57-1.80		32.49-2.15
R_work_/R_free_ c (%)	20.9/23.4		19.6/23.4
# of protein atoms	1907		1948
# of water molecules	168		87
hemes/monomer	0		1
Rmsd bond lengths (Å)	0.002		0.008
Rmsd bond angles (°)	0.566		1.192
PDB ID	3SZ6		3SIK
**Ramachandran plot statistics (%)**			
Preferred regions	210 (95.0)		238 (98.8)
Allowed regions	11 (5.0)		3 (1.2)
Outliers	0 (0)		0 (0)

a Values for the highest resolution shell are shown in parentheses.

b R_merge_ = Σ|I_hkl_−<I_hkl_>|/ΣI_hkl_, where I is the observed intensity for reflection hkl, and <I> is the mean intensity.

c R_work_ = Σ||F_o(hkl)_|−|F_c(hkl)_||/Σ|F_o(hkl)_|; R_free_ is calculated in the same way with 5–10% of reflections excluded from refinement.

The heme-binding pocket is enclosed primarily by a 3_10_-helix (residues Arg-54 to Tyr-58) on one side of the heme and a long β-hairpin (β7–β8) on the other. The 3_10_-helix is sometimes referred to as the “lip” because it seems to protrude over the heme-binding pocket [42]. The backbone of the 3_10_-helix in IsdX1 is fairly well-ordered even in the absence of heme. The integrity of the 3_10_-helix without heme could be due to the hydrogen bonding network between Ser-52, Ser-53, Arg-54 and Asn-56 and Met-55 from the helix making van der Waals contacts with residues in β4, β7, and β8.

**Figure 2:**
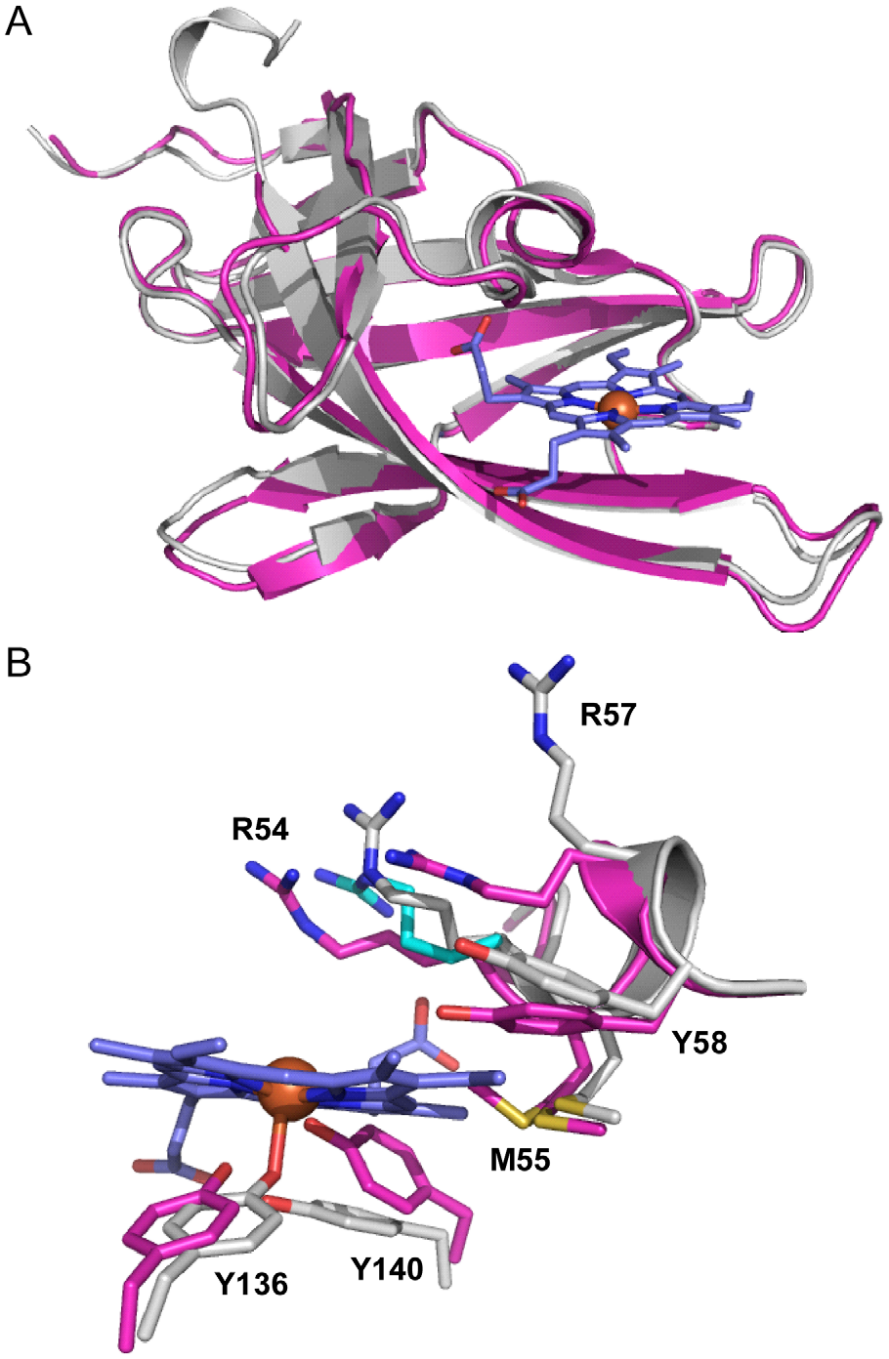
Superimposition of apo-IsdX1 and holo-IsdX1. (A) Ribbon representation of superimposition of apo-IsdX1 (pink) and holo-IsdX (grey). (B) Heme-binding pocket with stick representation of heme (carbon, blue and Fe, orange sphere), apo-IsdX1 residues (carbon, pink) and holo-IsdX1 residues (carbon, grey), and sulfur (yellow), nitrogen (blue) and oxygen (red). Arg-54 in the holo structure had two conformations and the alternate conformation has cyan carbon atoms.

The heme bound form of IsdX1 was solved to 2.15 Å by molecular replacement using the structure of apo-IsdX1 as the search model. Heme-iron coordination is achieved through Tyr-136, which is conserved among all heme-binding NEAT domain proteins (Figure S2, upper panel). The distance between the iron atom and the tyrosine oxygen ligand is 2.3 Å, which is typical for an Fe-O bond of NEAT domains (Figures 1B and 1C) [40]–[45]. The coordination bond to iron is stabilized by the conserved residue Tyr-140, which forms a hydrogen bond with the phenolate oxygen of Tyr-136. The aromatic ring of Tyr-140 further stabilizes the pyrrole ring through π-stacking. Both of these residues lie on a β-hairpin region, a conserved region in bacterial NEAT proteins that provides a structural platform for the heme [40], [42]. The least solvent exposed heme propionate forms a hydrogen bond with the hydroxyl group of Ser-53, as well as the backbone nitrogen of Arg-54. Additionally, the side chain NH1 group of Arg-54 from chain A also hydrogen bonds to the least solvent exposed heme propionate, and also to the hydroxyl group of Ser-53 (Figure 2A, B). However, this hydrogen-bonding network is not observed for the side chain of Arg-54 from chain B. The heme molecule is further stabilized by Tyr-58, that weakly π-stacks with the buried heme pyrrole ring. The other residues lining the heme-binding pocket are Arg-54, Met-55, Phe-59, Ile-84, Val-127, Ile-129, Ile-131, Ile-142 and Phe-144, which mainly form an aliphatic environment to accommodate the hydrophobic regions of the heme and its side chains.

### The role of the 3_10_-helix in heme binding

**Figure 3:**
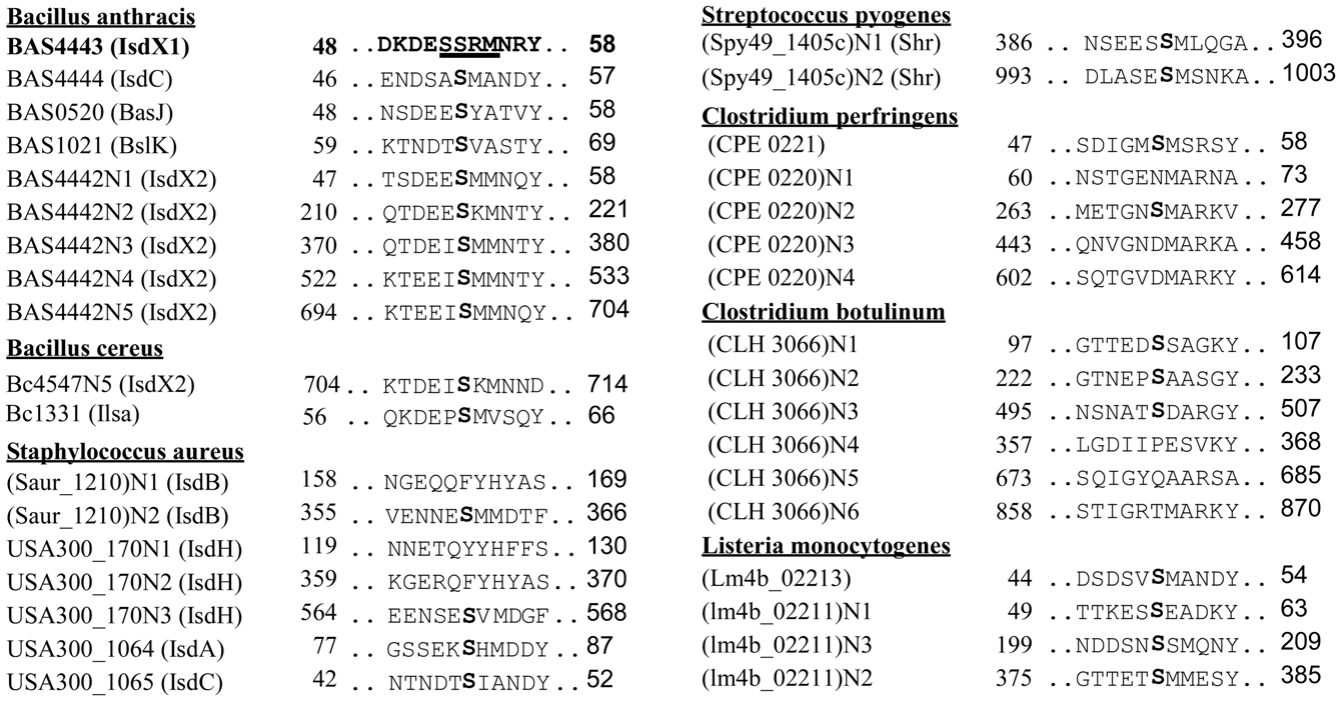
Alignment of the 3_10_-helix and adjacent residues of NEAT proteins in Gram-positive bacteria. ClustalW was used to align the amino acid sequences of the annotated NEAT proteins from the Gram-positive bacterial pathogens *Bacillus anthracis*, *Bacillus cereus*, *Staphylococcus aureus*, *Streptococcus pyogenes*, *Clostridium* (*botulinum* and *perfringens*), and *Listeria monocytogenes*. A serine (Ser-53 in IsdX1) is conserved in all nine NEAT domains from *B. anthracis* and in several NEAT domains from other Gram-positive bacteria. Although an arginine occupies position 54 in IsdX1, a methionine is commonly observed in related NEATs and may serve as a sixth axial ligand to the heme-iron, as described by Gaudin et al [62].

**Figure 4:**
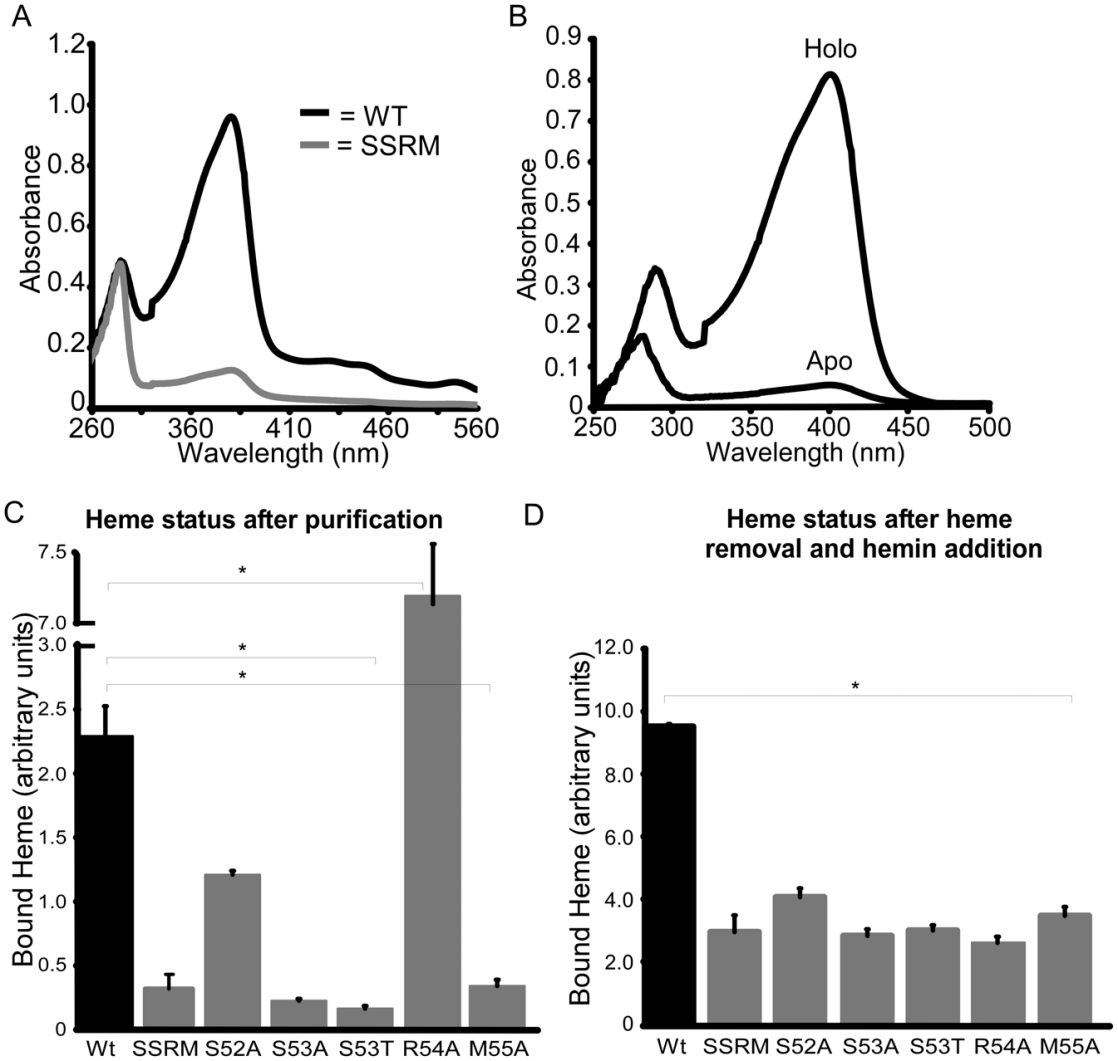
Functional role of the 3_10_-helix and adjacent residues: heme binding. (A) Ser-52, Ser-53, Arg-54, and Met-55 of IsdX1, designated SSRM, were each substituted to alanine and recombinant protein purified from *E. coli* as described in the *Experimental Procedures*. The absorbance properties immediately after purification from *E. coli* of wild-type (black) and SSRM (grey) IsdX1 were analyzed from 260–560 nm. (B) Recombinant IsdX1 was treated with low pH to remove co-purifying heme and the absorbance (250–500 nm) compared to the same preparation that was not acid treated. (C, D) Wild-type IsdX1, IsdX1-SSRM, or IsdX1 harboring mutations in Ser-52, Ser-53, Arg-54, or Met-55 were purified from *E. coli* and the heme content assessed by determining the ratio of the heme (399 nm) to protein (280 nm) absorbance (referred to as “bound heme”). In (C), the relative amount of associated heme is recorded following the purification of each IsdX1 variant from *E. coli*. In (D), all endogenous heme was removed from the preparations as described in (B) and apo-proteins incubated with 5 µM heme for 10 minutes at 25°C, followed by absorbance measurements. The absorbance value of a heme-only control (5 µM) was subtracted from all IsdX1 plus heme reaction readings. The values in (C) and (D) represent the mean and standard deviation of three independent experiments. The asterisk (*) means the differences were significant (p<0.05).

The location of the 3_10_-helix in the structures of the NEAT domains suggests this region is important for NEAT protein function, including heme binding, hemoglobin association, heme extraction and NEAT-NEAT heme transfer [41]–[43], [45]. There is some conservation in this region, as noted by Pilpa et al, with aromatic residues common in the equivalent positions of amino acids 54 and 58 for IsdX1 [46]. Interestingly, a serine residue (Ser-53 in IsdX1), which is immediately adjacent to the first residue of the helix (Arg-54), is well conserved in these NEATs, including every NEAT domain from *B. anthracis* (Figure 3, *bold*) [47]. To determine the role of this and adjacent residues in the ability of IsdX1 to bind heme, each amino acid in 52-SSRM-55 was changed to alanine and mutant proteins purified from *E. coli*. Whereas wild-type IsdX1 co-purified with a significant amount of endogenous heme from *E. coli*, IsdX1_(SSRM→AAAA)_ bound approximately 10-fold less heme after purification (Figure 4A, *quantitated in 4*C). Removal of the bound heme by organic extraction (Figure 4B) and quantitation of the Soret band intensity after titration of the apo protein with hemin confirmed the heme-binding defect of the mutant protein (Figure 4D).

To determine the residues responsible for this defect, single substitution changes in 52-SSRM-55 were generated and each purified mutant assessed for heme binding activity. As demonstrated in Figure 4C and D, mutation of Ser-52, Ser-53, or Met-55 decreased the ability of each of these mutant proteins to either co-purify with heme (Figure 4C) or bind exogenously added hemin (Figure 4D). The raw spectra for these mutants are illustrated in Figure S3. These effects are not due to gross disruption of IsdX1 secondary structure since the mutant proteins retained a similar overall β-sheet content as the wild-type protein when assessed by far-UV circular dichroism (Figure S4). Further, the heme binding site seems to be somewhat intolerant of even small structural changes, since substitution of Ser-53 to a threonine, which differs only in the length of the side chain (extra methyl group), also abolished the interaction with heme (Figure 4 C,D). Interestingly, mutation of Arg-54 led to a purified IsdX1 preparation with a Soret band approximately three times greater than wild-type protein (Figure 4C). The high heme content in this sample was confirmed using the pyridine hemochrome method, which demonstrated the molar amounts of heme, on average, were 75–90% the molar concentration of protein, suggesting binding was stoichiometric for this preparation (data not shown). However, upon removal of the heme and incubation of R54A with hemin, very little heme bound the protein, despite a far-UV spectrum indistinguishable from wild-type protein (Figure 4D, Figure S4). In the structure of apo and holo-IsdX1, the side chain of Arg-54 shifts 2.5–2.8 Å to accommodate the heme (Figure 2B). This suggests placement of a less bulky residue (alanine) in place of Arg-54 may allow heme access to the heme-binding site, but potentially only during translation of the protein when partially unfolded. Regardless of the exact reason for this, these results demonstrate residues in and around the 3_10_-helix are involved in the binding of heme to IsdX1.

### 3_10_-helix residues and heme stabilization

In an attempt to provide a more quantitative assessment of the importance of each residue in the 3_10_-helix to the stabilization of bound heme, we measured the rates of heme dissociation from wild-type and mutant IsdX1 proteins. Each protein was purified from *E. coli* as described in the Materials and Methods, reconstituted with hemin, and holo-protein purified away from unbound hemin by gel filtration chromatography. The rate of heme dissociation was then assessed by mixing holo-IsdX1 preparations with excess H64Y/V68Y apo-myoglobin (Mb), a mutant globin with a high heme affinity (K_d_∼10^−12^ M) and very low rate of heme dissociation [30], [48], [49]. The dissociation rate constant of heme loss from IsdX1 can be determined by measuring the spectral changes that occur with time as released heme is scavenged passively by the apo-Mb reagent.

**Figure 5:**
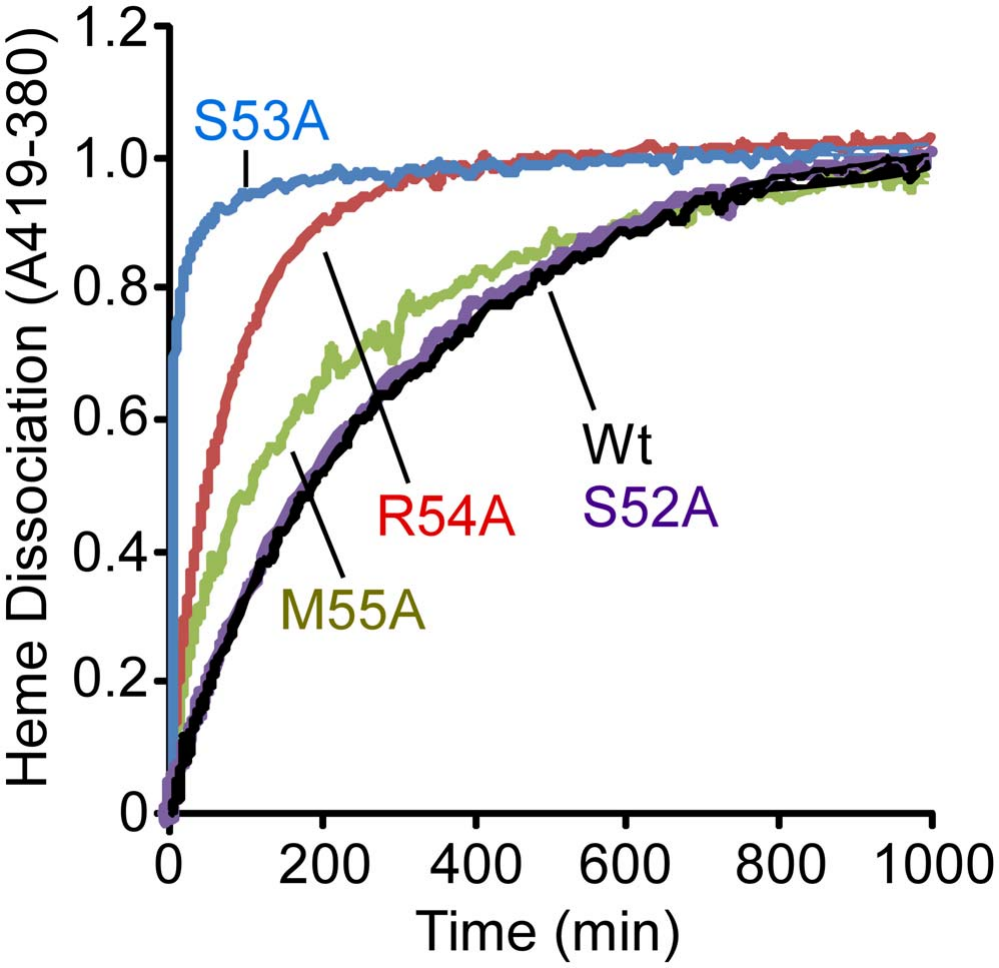
Heme dissociation kinetics for wild-type and mutant IsdX1. Wild-type or mutant (S52A, S53A, R54A, or M55A) IsdX1 were purified from *E. coli* and endogenous heme removed as described in the Materials and Methods. Proteins were re-constituted with heme, excess heme removed by gel filtration chromatography, and holo proteins (1 µM) mixed with H64Y/V68F apo-Mb (26 µM) at 25°C in PBS, pH 7.4. Time courses for hemin dissociation the IsdX1 variants were determined by measuring the difference between in the increase in absorbance at 419 nm (peak for holo H64Y/V68F holo-Mb) and the decrease in absorbance at 380 nm (strong absorbance by holo-IsdX1).

As observed in Figure 5, IsdX1 containing mutations in Ser-53, Arg-54, and Met-55 all lose heme significantly faster than wild-type IsdX1, with S53A showing rates of heme loss that were greater than 400 times faster than the wild-type IsdX1 (see Table 2 for rates). Interestingly, S52A showed comparable rates of heme dissociation to that of wild-type, despite its apparent poor heme binding ability at equilibrium (Figure 4D). The best explanation for this is that while its rate of heme loss may be unaffected, its rate of heme association in the absence of hemoglobin may be poor. Taken together, the data are consistent with Ser-53 and Arg-54 of the 3_10_-helix playing a substantial role in stabilizing the bound heme in IsdX1.

**Table 2:** Rate constants for heme dissociation from IsdX1 variants.

IsdX1 variant	Rata constant (min^−1^)	Halftime (min)^a^
Wild-type	k = 0.0034	190
S52A	k = 0.0034	190
S53A^b^	k_1_ = 1.4 (70%), k_2_ = 0.03 (30%)	1
R54A	k_1_ = 0.042 (30%), k_2_ = 0.0035 (70%)	110
M55A	k_1_ = 0.077 (20%), k_2_ = 0.01 (80%)	47

a The halftime is defined as the amount of minutes for one-half of the heme to dissociate from IsdX1.

b For S53A and R54A, the dissociation curves are best described by two phases, each with a single rate constant. The percentages indicate the proportion of the total population giving that particular rate.

### The role of the 3_10_-helix in hemoglobin association

**Figure 6:**
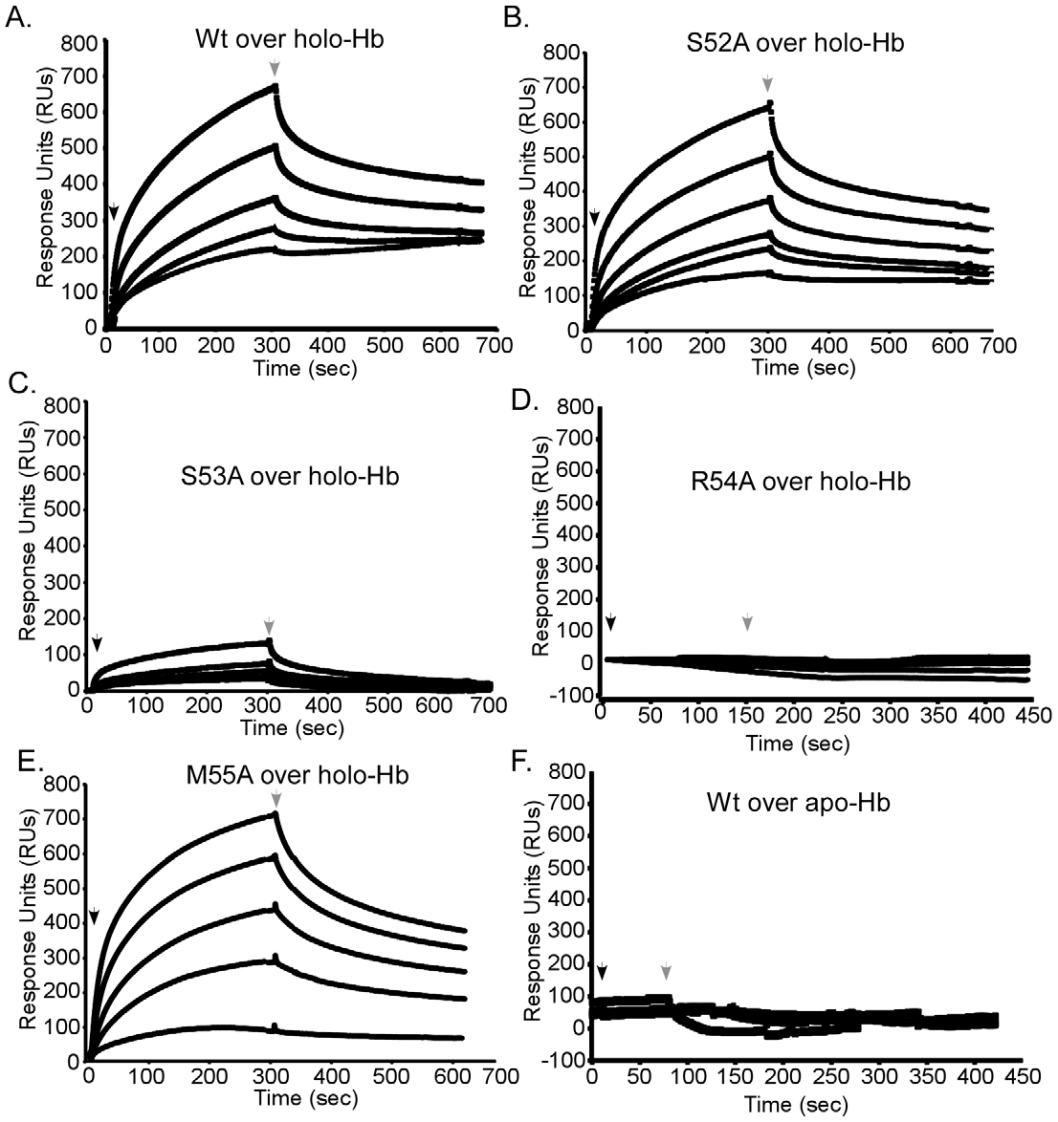
Functional role of the 3_10_-helix and adjacent residues: hemoglobin association. Wild-type (A), S52A (B), S53A (C), R54A (D), or M55A (E) IsdX1 were infused at 100, 200, 300, 350, or 500 nM over holo or apo-hemoglobin (wild-type only, panel F) coupled to a CM5 chip and response units recorded over 800 seconds. The dissociation constants (in nanomolar) were as follows: wild-type = 15.0±0.07, S52A = 14.0±0.17, S53A = 139.0±2.5, R54A = 5, 500±1, 800, and M55A = 18.0±0.4. Due to the weak response of R54A, the *K_D_* was calculated from response curves using concentrations approximately 100 times that injected for the wild-type protein. All other dissociation constants represent the mean and standard deviation of three independent measurements for the injection of 300 nM (final concentration) IsdX1 (χ^2^<than 1.0). Hb = hemoglobin.

The position of 52-SSRM-55 extending over the heme-binding site implies these residues may also be involved in the direct interaction with hemoglobin. To test this hypothesis, the association of each mutant with holo-hemoglobin was investigated by surface plasmon resonance spectroscopy. Apo forms of wild-type or mutant IsdX1 were infused over holo-hemoglobin covalently coupled to a carboxy-methyl chip and the kinetics of binding recorded by quantifying the change in response units (RUs) with time. Whereas similar responses were observed for wild-type, S52A, and M55A IsdX1, a significantly lower response was observed when S53A or R54A were infused over holo-hemoglobin (Figure 6, compare panels A, B, E to panels C, D). Indeed, estimations of the dissociation constants (*K_D_* – Figure 6 legend) from the association and dissociation phases of the response curves indicates that the S53A and R54A mutants, when compared to wild-type IsdX1, display an approximately 10 and 386-fold increase, respectively, in *K_D_*, signifying a lower affinity for hemoglobin. There was no interaction of the wild-type or mutant IsdX1 proteins with apo-hemoglobin, which infers binding is dependent on a heme-bound conformation of hemoglobin or is facilitated by interactions with the solvent exposed heme propionates in holo-hemoglobin (Figure 6F). These results indicate Ser-53 and Arg-54 are important in the interaction of the IsdX1 hemophore with holo-hemoglobin and collectively suggest the processes of heme binding and hemoglobin association in NEATs are coupled for some residues but not others.

### The role of the 3_10_-helix in hemophore biology

**Figure 7:**
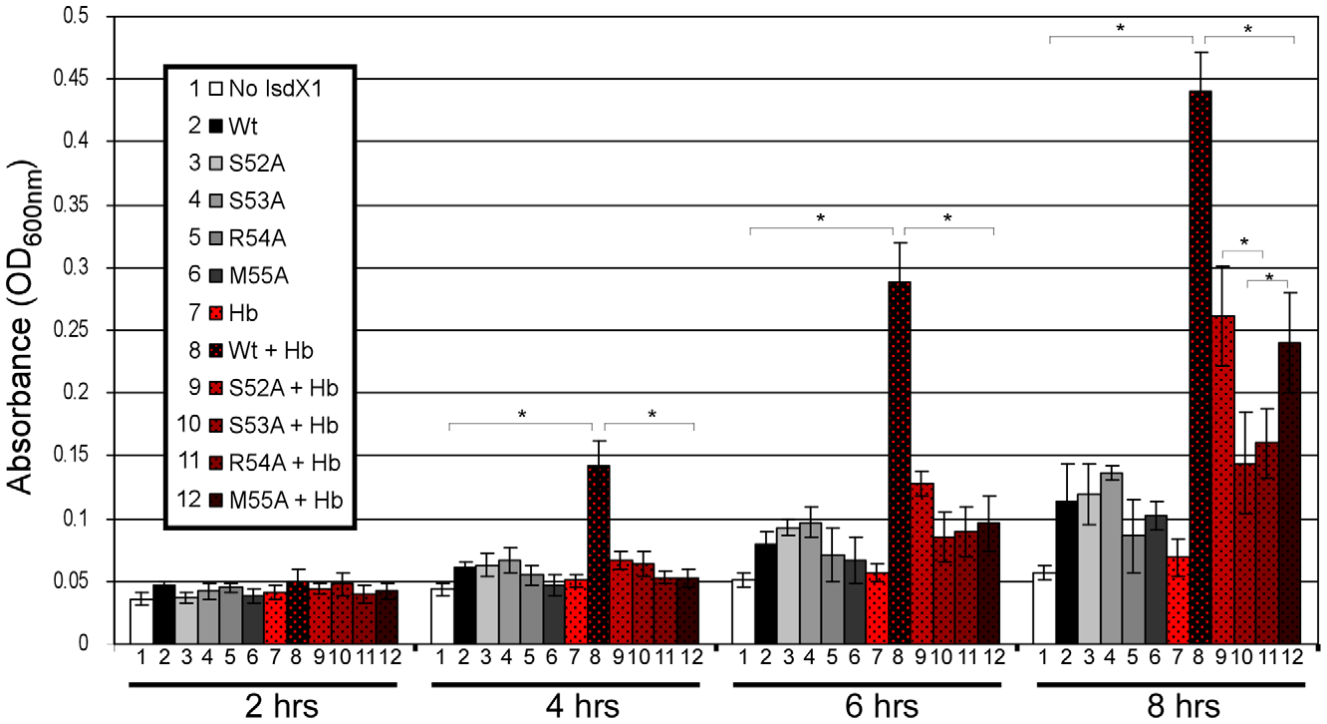
Functional role of the 3_10_-helix and adjacent residues: hemophore and NEAT-domain biology. Purified wild-type or mutant IsdX1 were added to a final concentration of 1 µM to hemophore-deficient (Δ*isdX1*, Δ*isdX2*) *B. anthracis* Sterne 34F2 grown in iron-chelated RPMI with or without hemoglobin (10 µM) and the OD_600_ recorded at 2, 4, 6, and 8 hours. The results represent the mean and standard deviation of three independent experiments. The asterisk (*) means the differences were significant (p<0.05). Hb = hemoglobin.

IsdX1 and IsdX2 promote the growth of *B. anthracis* on hemoglobin as the sole source of iron [26]. To determine the functional contribution of the 52-SSRM-55 helix towards heme scavenging activity, we tested the ability of wild-type and mutant proteins to rescue a hemophore-dependent growth defect of a *B. anthracis* strain (Δ*isdX1*, Δ*isdX2*) lacking both hemophores grown in iron-deficient media with or without hemoglobin. Although little growth is observed in the absence of hemophore, the addition of wild-type IsdX1 to cultures with hemoglobin led to a 3 to 8-fold increase in growth (Figure 7, *compare 7 to 8*, *from 4 to 8 hrs*). This enhancement of growth is not due to heme or iron contamination of the protein preparation since only a marginal increase in growth was observed in the absence of hemoglobin (Figure 7, *compare 8 to 1,2*). Interestingly, whereas Δ*isdX1*, Δ*isdX2 B. anthracis* supplemented with S52A or M55A IsdX1 provided intermediate growth (Figure 7, *compare 9,12 to 3,6 at 8 hrs*), the level of replication in the S53A and R54A IsdX1 supplemented cultures was similar to the S53A/R54A-only controls, suggesting these proteins are unable to rescue a Δ*isdX1*, Δ*isdX2*-dependent growth defect on hemoglobin as the sole iron source (Figure 7, *compare 10,11 to 4,5 at 8 hrs*). Taken together, these results indicate Ser-53 and Arg-54-mediated association of IsdX1 with hemoglobin is important for heme scavenging in iron-limiting environments and provides the first experimental demonstration of the biologic function of this dynamic region in NEAT proteins in growing cells.

## Discussion

Here, we report (i) the crystal structure of the apo and holo forms of a Gram-positive hemophore, (ii) the structure of a non-staphylococcal NEAT protein, (iii) that residues in and adjacent to the 3_10_-helix contribute to the binding of the heme-iron in this hemophore, (iv) that the processes of heme binding and hemoglobin association can be delineated, and (v) that both optimal heme and hemoglobin binding are necessary for full hemophore activity for growing bacilli. Thus, these studies extend our knowledge of the molecular mechanism of NEAT protein function and provide evidence that abolishing a functional interface between the NEAT domain and hemoglobin can slow bacterial growth in iron-limiting environments.

Research into bacteria hemophores is a growing field, and several hemophores have been discovered [10], [16], [50], [51]. The most well-documented secreted hemophore is that of HasA from the Gram-negative pathogen *S. marscescens* [52]. Although functionally similar, it is likely that IsdX1 and HasA resulted from convergent evolution, as there is little structural similarity between the two proteins. Whereas HasA obtains heme from hemoglobin through a passive mechanism that does not seem to require a physical interaction, IsdX1 binds hemoglobin directly, an event that likely facilitates heme transfer [15], [24], [26]. Both proteins transfer the bound heme to their cognate cell surface receptors (IsdC for IsdX1 and HasR for HasA) through direct engagement. However, the IsdX1-IsdC interaction is dependent on the hemophore being heme loaded and is transient [30]. This contrasts with the HasA-HasR interaction, where both the apo and holo forms of HasA bind with similar affinities and to the same site on HasR [15], [53]. More recently, several secreted hemophores have been reported. HmuY, from *P. gingivalis*, binds heme and may deliver its heme to the surface receptor HmuR [54], [55]. Also in *P. gingivalis*, the recently described HusA is a heme-binding protein that is needed for growth under conditions of heme limitation [12]. A putative secreted hemophore from *Mycobacterium tuberculosis* has been characterized with a proposed heme-binding site consisting of one Tyr and two His, which has a similar heme-binding structural motif to that of HasA [56]. However, the overall fold of the Mtb hemophore is structurally diverse in comparison with both HasA and IsdX1 folds, and it was postulated that the Mtb hemophore may also be a product of convergent evolution [56].

Recent work has shed new insights into how Gram-positive bacteria acquire heme from mammalian hosts [57], [58]. The central structural unit is the NEAT domain, a protein module that mediates heme acquisition and import at the bacterial surface [26], [29], [31], [41], [57]–[61]. If NEAT proteins are to be targets for the development of anti-infectives, the molecular determinants of their mechanism of action need to be defined. In this context, the study of IsdX1 is particularly appropriate because this protein contains the only known NEAT domain that contains all the activities associated with hemophore activity (heme and hemoglobin association, heme extraction from hemoglobin, and heme transfer to receptors) [26]. Thus, the study of IsdX1 allows for insights into the relationship between heme coordination, hemoglobin binding, and heme extraction from mammalian globins.

To gain insights into these activities, we solved the 3-dimensional structure of apo and holo IsdX1. The overall structure is similar to the structures of the staphylococcal NEAT domains of IsdH (NEAT 1/3) [40], [44], IsdA [41], IsdC [42], [43], and IsdB (NEAT 2) [62], with three common features: (i) an immunoglobulin-like fold arranged into eight β-sheets, (ii) a small 4–5 residue 3_10_-helix extending over the heme-binding pocket, and (iii) two anti-parallel β-sheets that house a conserved tyrosine (Tyr-136 in IsdX1) which coordinates the heme-iron (Figure S2A). However, surface charge calculations indicate a charge distribution on IsdX1 quite distinct from the other NEAT domains, including a net positively charged region near the 3_10_-helix of the heme pocket (Figure S1). It is not known if this difference relates to the fact that IsdX1 acts extracellularly after secretion, as opposed to the staphylococcal NEAT proteins that are covalently anchored to the cell wall. Of note is the finding that the staphylococcal homolog of the putative receptor for IsdX1 (IsdC) contains overall anionic character in this region, a feature which leads one to postulate that opposing charges may partially dictate the association between these two proteins upon heme transfer.

The structures of apo-IsdX1 and holo-IsdX1 are highly similar with RMSD of 0.51 Å. However, superimposition of both apo and holo structures highlight subtle residue sidechain conformational differences surrounding the heme binding site. This finding suggests residues in the 3_10_-helix may partially stabilize the heme-free form (discussed below), a requirement of this hemophore immediately after secretion into host tissues. To accommodate the heme molecule, there is a shift in the residue sidechains of Arg-54 (2.5–2.8 Å), Arg-57 (7.3 Å), Tyr-58 (2.1 Å), Tyr-136 (1.6 Å) and Tyr-140 (2.6 Å), away from the center of the heme binding pocket. Additionally, there is observable electron density for two side chain conformations for Met-55 within the apo structure, where one conformation is pointing toward the center of the heme binding pocket and the other away into the interior of IsdX1. Met-55 in the holo structure has its side chain pointing into the interior of the protein and makes hydrophobic contacts with the heme pyrrole ring. In contrast, there is no conformational change observed for either Ser-52 or Ser-53 between the holo and apo structures.

It is interesting to note that within the holo-IsdX1 structure, crystal lattice packing interactions occur through a heme-mediated protein interface between two holo-IsdX1 molecules from adjacent asymmetric units (Figure S5A, B). Two heme molecules from adjacent subunits planar stack upon one another (inter-iron distance of 5.7 Å) and the crystallographic interface is further stabilized by two hydrogen-bonds; one from the NH2 group of Arg-54 from Chain A to the most solvent exposed heme propionate from Chain B and the second from the NH1 group from Arg-57 from Chain B to the hydroxyl group of Asn-135 on the adjacent molecule. The apo-IsdX1 structure also has a similar protein interface near the vicinity of the heme-binding sites of adjacent monomers; however due to the lack of heme, there are more protein interactions between loop regions surrounding the active site (Figure S5A,B). We believe the heme stacking interaction, while caused by crystal packing, may reflect how IsdX1 transfers heme to downstream NEAT domain proteins such as *B. anthracis* IsdC. Indeed, Grigg et al (published during review of this paper) proposed that inter-NEAT domain interactions between *S. aureus* IsdB and IsdA may occur along the heme-mediated crystal symmetry interface based on *in silico* docking predictions [63]. Moreover, an NMR analysis by Villareal et al (also published during review) of the interaction between *S. aureus* IsdC and IsdA demonstrated that in the IsdC-IsdA complex the proteins are pseudosymmetrically arranged through a 180° rotation that is coplanar with the heme plane, which is identical to the symmetry relationship between IsdX1 monomers from adjacent asymmetric units [64]. Thus, our data also lend experimental support to these models.

With respect to the residues in and around the 3_10_-helix of IsdX1, several interesting properties can be gleaned from this study. First, amino acids in this region contribute to the stabilization of heme, as demonstrated by the fact that the substitution of Ser-52, Ser-53, and Met-55 to alanine abrogated the ability of IsdX1 to attain heme from *E. coli* lysates as well as bind pure hemin when incubated with the apo protein. Mutation of Arg-54 led to significantly higher amounts of heme co-purifying with IsdX1. However, removal of this heme and assessment of hemin binding yielded a protein unable to subsequently coordinate heme. We cannot definitively explain this result, other than to propose that it is possible that while being synthesized in *E. coli*, the partially unfolded IsdX1 binds the heme and then folds thereby enclosing upon the iron-porphyrin. Upon heme removal, the resulting apo protein, potentially highly ordered, now becomes restricted and does not allow heme to access the binding pocket. Interestingly, this was not observed in an IsdA variant harboring an alanine substitution in the equivalent position (His-83) [41]. It was proposed this position in IsdA and IsdC (Ile-48) sterically hindered access to the sixth coordination position of the heme-iron. Indeed, cyanide and azide, two ligands often used to probe accessibility to the sixth position, did not bind IsdA or IsdC [42], [65]. The ability of the *E. coli* form of R54A to associate with more heme while expressed in *E. coli* may provide experimental support for this hypothesis, with Arg-54 sterically blocking access to Try-136 in the wild-type protein.

Second, mutation of this region decreases hemoglobin association; however, differential effects are observed. Substitution of Ser-53 and Arg-54 with alanine significantly reduced binding to hemoglobin, with R54A yielding the largest effect (greater than 300-fold). However, mutation of Ser-52 and Met-55, the two residues flanking Ser-53 and Met-54, produced hemoglobin-binding affinities similar to the wild-type protein. These findings support a model by which Arg-54, being rather forward in its location over the heme-binding site, provides initial contact with hemoglobin, perhaps stabilizing the initial interaction. The engagement of Arg-54 with hemoglobin may “peel” the side chain of this residue away from the heme-binding site, thus removing the steric block observed for residues in this position for other NEAT proteins. Further support for this hypothesis is that the hemoglobin-binding NEAT domain, IsdB-N2, has a Met in the same position as Arg-54, and the crystal structure of IsdB showed an alternate conformation of Met coordinated with heme-iron, whereby the authors suggest that this Met might be involved in heme transfer. If alternate conformations of Arg-54 are sampled, one conformation has the NH1 group of Arg-54 coordinated to heme-iron similar to IsdB (Figure S2, lower panel). Thus, Arg-54, by virtue of its unique orientation in the heme-binding pocket, may mediate several processes related to heme extraction from hemoglobin. In essence, this residue may function as a “molecular placeholder” by initially binding the heme during the first step of transfer. In support of this hypothesis, there is evidence that arginine can assist in heme binding in other systems [66]. Ser-53, whose side chain is directed towards the heme-binding site, would next be free to hydrogen bond with a heme propionate, thereby strengthening the IsdX1-hemoglobin interaction.

Once Ser-53 engages the heme, Tyr-136 of IsdX1, from the opposite side of the heme, can now displace hemoglobin's His-iron coordination to become the fifth axial ligand of IsdX1, with Tyr-140 providing additional strength for this coordination by H-bonding to the phenolate of Tyr-136. In addition to Tyr-136, the heme is also secured in the binding pocket by Ser-53 and Arg-54, since these residues show the highest rates of heme loss when mutated. Ser-52, whose mutation does not lead to greater heme dissociation, may also assist in drawing the heme into the heme-binding pocket but once the heme is in, does not contribute much to its stabilization. This hypothesis aligns well with the observation that the side chain of this residue sticks out into the solvent while the side chain of its neighbor, Ser-53, points into the heme-binding pocket.

During review of our manuscript, Kumar et al described the first ever co-crystal of a NEAT protein (the first NEAT domain of IsdH from *S. aureus*) in complex with the alpha-chain of hemoglobin [67]. The structure reveals several stabilizing contacts between the 3_10_-helix of IsdH and hemoglobin, thereby confirming our predictions for the role of this helix in direct association with hemoglobin and heme scavenging. Perhaps most important is a hydrogen bond between a serine in IsdH NEAT 1 (Ser-130) with Lys-11 on hemoglobin. This serine is just 5 residues downstream of what would be the position of Ser-53 in IsdX1, leading us to speculate that the lack of functional interaction with hemoglobin observed upon mutation of this residue is because a key hydrogen bond is severed in the IsdX1-Hb interaction.

Whether or not the function of the 3_10_-helix is confined only to heme acquisition from hemoglobin remains to be determined. For example, Grigg et al found the conserved coordinating tyrosine in the first NEAT domain of IsdA (the equivalent of Tyr-136 in IsdX1), and not residues in the 3_10_-helix, played a role in NEAT to NEAT heme transfer [63]. In contrast, Villareal et al found that mutation of a single 3_10_-helix residue in *S. aureus* IsdA did affect NEAT to NEAT heme transfer; however, this mutation was coupled to another mutation elsewhere in the protein that was also believed to mediate NEAT-NEAT binding [64]. Thus, more work is required to determine if the 3_10_-helix also functions in downstream heme transfer processes.

Although this model of hemoglobin and heme binding is consistent with available data, we cannot rule out that there are additional residues outside of the 3_10_-helix that participate in this process. Indeed, as demonstrated by Pilpa et al [46] for one of the staphylococcal hemoglobin receptors (NEAT 1 of IsdH) [68], [69], amino acids distal to this position (on the β3–β4 loop), also seem to be important for hemoglobin association. However, NEAT 1 of IsdH does not bind heme, and instead requires a third NEAT domain (NEAT 3), to acquire and stably bind the heme from the IsdH NEAT 1-hemoglobin complex. Thus, the data suggests residues in and around the 3_10_-helix in IsdX1 evolved to bind both heme and hemoglobin, as well as those that are not interdependent, in order to provide several functionalities (hemoglobin binding coupled to heme extraction) within a single NEAT domain.

Finally, the exogenous addition of recombinant IsdX1 to culture restored the growth of a hemophore-deficient strain of *B. anthracis* on hemoglobin, suggesting all the information necessary for hemophore activity is contained within its amino acid sequence. The partial restoration of growth observed by S52A and M55A may be due to the ability of these mutants to still bind heme, albeit poorly, after association with hemoglobin. Although S53A and R54A bind heme at similar levels as S52A and M55A, it would seem their much lower affinity for hemoglobin precludes them from acquiring any heme in this assay (or alternatively, does not promote the release of enough heme from hemoglobin), meaning no or little free heme is available for transfer (or sequestration) at the bacterial surface. Because mutation of Ser-52, Ser-53, Arg-54, and Met-55 results in poor heme-binding activity, it is difficult to biochemically determine the exact role of these residues in the ability of IsdX1 to extract heme from hemoglobin. However, it is clear from the SPR and growth studies that mutations that effect the association with hemoglobin significantly compromises hemophore activity. Performing an identical experiment with a single mutant (Δ*isdX1*) strain did not yield a significant enough phenotype to evaluate these mutants for function (data not shown), likely because IsdX1 and IsdX2 are functionally redundant [26]. Taken as a whole, these experiments highlight the biological role of the 3_10_-helix in NEAT protein function and hemophore biology and provide direct evidence that the mechanistic extraction and binding of heme from hemoglobin are important for *B. anthracis* replication in low-iron environments.

Although attempts to develop a universal inhibitor of heme uptake is likely to be challenging, the seemingly conserved functional properties shared by distinct NEAT domains does offer the prospect of targeting these processes in select Gram-positive bacteria. As we make advances in our understanding of NEAT mechanism of action, the elucidation of additional structures, an understanding of side chain chemistry in transfer reactions, and the identification of factors that drive ligand binding specificity, will all be required for the creation of novel anti-infectives that prevent iron-porphyrin uptake in these pathogenic bacteria.

## Materials and Methods

### Bacterial strains, reagents, and mutagenesis

DNA encoding for amino acids 26–146 of IsdX1 were amplified from the genome of *B. anthracis* strain Sterne 34F2 using PCR and primers containing EcoRI and BamHI restriction sites [70]. Amplified DNA was digested, ligated into pGEX2TK, and pGEX-IsdX1 transformed into *E. coli* BL21 for the expression of IsdX1 as a glutathione-S-transferase (GST) fusion protein as described [26], [29]. For the purification of IsdX1 used to generate the holo-protein crystals, DNA encoding residues 27–152 was cloned into pET28a (Novagen) encoding a fusion protein of IsdX1 with a His_(6)_-tag using NheI and NotI restriction enzyme sites. The 26–146 (apo) and 27–152 (holo) protein constructs were chosen because they yielded crystals that diffracted with the highest resolution. Site-directed mutagenesis of IsdX1 was performed on the 26–146 IsdX1 construct using QuikChange (Stratagene, Santa Clara, CA) according to the manufacturer's instructions. After DpnI digestion of reaction mixtures, DNA was transformed into *E. coli* BL21 and the resultant plasmid clones sequenced to confirm the presence of the mutation [71], [72]. All *E. coli* strains were grown in Luria-broth (LB) supplemented with 50 µg/mL ampicillin (Fisher Scientific, Waltham, MA) except for the pET28a construct that was supplemented with 30 µg/mL kanamycin (Fisher).

### Purification of proteins for activity studies

Wild-type and mutant IsdX1 proteins were purified by GST-affinity chromatography as previously described [26], [29]. Briefly, 50-mL of overnight cultures of *E. coli* BL21 containing wild-type or mutant p*gst-isdX1* were inoculated into 2-L of LB with ampicillin and rotated at 250 rpm at 37°C. After 2 hours, isopropyl- β-D-thiogalactopyranoside (IPTG - 1.5 mM) was added and cultures grown for an additional 2 hours. Bacteria were centrifuged at 6,000×*g* for 8 min, resuspended in phosphate buffered saline (PBS - 137 mM NaCl, 2.7 mM KCl, 10 mM sodium phosphate dibasic, 2 mM potassium phosphate monobasic, pH 7.4), and cells lysed by French press. The supernatant was obtained by centrifugation at 30,000×*g* for 15 min and filtered through a 0.45-µm pore-size cellulose filter. Lysates were next applied to 1-mL of glutathione-sepharose (GE Healthcare, Piscataway, NJ), washed with 40-mL of PBS, and bound protein incubated with thrombin (100 units, GE Healthcare) for 3 hours at 25°C. GST-free IsdX1 preparations were next incubated with 200-µL of aminobenzamidine sepharose (Sigma, St. Louis, MO) to remove thrombin. To remove endogenous heme, preparations were treated with HCl (final pH of 2.0) and methyl ethyl ketone was added to separate heme (organic layer) from IsdX1 (aqueous layer) as described [73]. Protein concentrations were either determined by UV/vis spectroscopy, the bicinchoninic acid method (Pierce, Rockford, IL), or by SDS-PAGE [74]. All protein preparations were stored at −20°C.

### Purification of proteins for crystallography

apo-IsdX1 - To obtain an IsdX1 preparation for crystal seeding, IsdX1 was expressed and bound to glutathione-sepharose as described above [26], [29]. Bound protein was then cleaved from the column with Factor-XA (10 units, Amersham Biosciences) in 1 mM CaCl_2_, 100 mM NaCl, 50 mM Tris pH 7.9 for 16 hours. Heme was removed as described above and IsdX1 further purified by cation exchange chromatography using a Mono S column (GE Healthcare) and gel filtration chromatography using a Superdex 200 column (GE Healthcare). Selenomethionine-substituted protein was produced by inhibiting methionine biosynthesis and purified as above.

Holo-IsdX1 - IsdX1 was transformed into BL21-Gold (DE3) cells and grown at 37°C in LB medium containing 30 µg/mL kanamycin. Protein expression was induced when cells reached OD_600 nm_ of 0.8 by the addition of 1 mM IPTG and cells harvested after 4 hours by centrifugation at 5, 100 rpm for 20 minutes, followed by resuspension in 50 mM Tris, pH 7.4, and 350 mM NaCl. Cells were next lysed by sonication after addition of egg hen lysozyme (5 mg, Sigma) with phenylmethylsulfonyl fluoride (40 µM, Sigma) and the cell lysate centrifuged at 14, 000 rpm for 20 minutes. After addition of 400 µL Proteoblock protease inhibitor cocktail (Fermentas), the supernatant was loaded onto a Ni^2+^-charged HisTrap column (GE Healthcare) and eluted with a linear imidazole gradient (between 100–250 mM imidazole). Fractions containing IsdX1 were identified by SDS-PAGE, pooled and concentrated using a Centricon centrifugal concentrator (Millipore). Further purification of IsdX1 was achieved by running the protein over an S75 gel filtration column (GE Healthcare) equilibrated with 50 mM Tris pH 7.4, 150 mM NaCl, which yielded nearly 100% homogeneous protein. Cleavage of the His_(6)_-tag was conducted in cleavage buffer (50 mM Tris pH 7.4, 150 mM NaCl, 10 mM CaCl_2_) by adding 1 mL of thrombin-agarose suspension (Sigma) to the protein, followed by removal of thrombin-agarose on a glass frit. IsdX1 was then run over an S75 gel filtration column equilibrated with 50 mM Tris pH 7.4, 150 mM NaCl to separate IsdX1 from the His_(6)_-tag.

### Crystallization and structure determination

apo-IsdX1 - Purified IsdX1 in buffer A [10 mM MES pH 6.6, 200 mM NaCl] crystallized at room temperature using the hanging drop vapor diffusion method and a reservoir solution of 0.1 M citric acid pH 3.5 and 2 M NaCl. Crystals were frozen in N_2_ (*l*) following cryoprotection with the reservoir solution containing 16% glycerol. Data were collected to 1.8 Å at the Structural Biology Center beamline 19-BM at the Advanced Photon Source (APS), Argonne National Laboratory (ANL), and processed using HKL2000 [75]. SeMet-IsdX1 crystallized with a reservoir solution of 0.1 M citric acid pH 3.5 and 3.3 M NaCl and was frozen as above. Data were collected to 2.1 Å at the Life Sciences Collaborative Access Team (LS-CAT) beamline 21-ID-D at the APS, and processed with XDS and scaled using SCALA (Table 1) [76]. The structure was determined by the single-wavelength anomalous dispersion method using the anomalous scattering from two selenium atoms in the asymmetric unit cell with the program PHENIX [77]. The model was built automatically with PHENIX and manually with Coot[78] and refined with PHENIX.

Holo-IsdX1 - Approximately 4 mg of heme were dissolved in 0.4 mL of ice cold 0.1 M NaOH and vortexed periodically. After 30 minutes, 0.4 mL of 1 M Tris, pH 7.4 was added to the solution. The solution was subsequently centrifuged for 10 minutes at 4°C at 13,000 rpm. The heme solution was then diluted with 50 mM Tris, pH 7.4, 150 mM NaCl and centrifuged again at 5, 100 rpm to remove any heme aggregates. Final concentrations were determined using ε_385_ = 58.44 mM^−1^ cm^−1^. Heme solutions were used within 12 hours.

Holo-protein was reconstituted by slowly adding 1.5-fold excess heme to IsdX1 preparations in small increments. The UV/vis absorption spectrum was recorded after each addition of heme and saturation with heme was evident by the shift in the Soret maximum from 399 nm to a shorter wavelength. After 1-hour incubation at room temperature, excess heme was removed using an S200 gel filtration column (GE Healthcare) and the protein collected in 1-mL fractions. The UV/vis absorbance spectrum for each fraction was recorded and those with a Soret peak maximum at 399 nm and Abs_399_/Abs_280_ ratios greater than 3 were pooled. Protein concentrations were measured using the modified Lowry assay (Pierce, Rockford, IL). The extinction coefficient for the Soret peak at 399 nm was determined to be equal to 100 mM^−1^ cm^−1^ by the pyridine hemochrome assay and used for hemoprotein concentration determination [79]. Holo-IsdX1 was concentrated to 100 mg/mL in 50 mM Tris pH 7.4, 150 mM NaCl for crystallization trials.

Crystals of holo-IsdX1 were grown by the hanging drop, vapor diffusion method against a reservoir containing 0.1 M SPG buffer, pH 9.0 and 25% polyethylene glycol 1500 with crystallization drops containing 0.25 µL of protein to 0.25 µL of reservoir solution. Crystal growth was observed within 24 hours. The crystal was passed through a 1∶1 v/v solution containing the reservoir solution and glycerol for cryoprotection and the crystals harvested under cryoconditions. The diffraction data was collected at 77 K on beamline 9-2 at Stanford Synchrotron Radiation Lightsource. Two data sets were collected, a native set at 1.0 Å and a Fe-SAD set at the iron absorption edge (1.738 Å). Images were indexed, integrated and reduced using the HKL2000 suite resulting in a 99.1% complete dataset to 2.15 Å resolution for the native set and a 98.2% complete dataset to 2.3 Å resolution for the Fe-SAD set. The unit cell dimensions are equal to 65.1 Å×65.1 Å×74.4 Å in the space group P4_3_ with two molecules of holo-IsdX1 per asymmetric unit (Table 1). The initial phases were calculated using Phaser [80] with apo-IsdX1 as the search model and the resulting electron density map was subjected to automated and manual model building procedures through a combination of PHENIX.autobuild [77], PHENIX.refine [77] and Coot [78]. Subsequently, two heme-iron sites per asymmetric unit were detected from the anomalous iron signal using SHELXC/D/E [81]. This additional phase information was combined with the molecular replacement model phases through SIGMAA and DM in CCP4i [82], which improved the resulting electron density map for a final round of refinement. Programs from the CCP4 package [82] as well as Phenix [77], Pymol [83], and Coot [84] were used to analyze the stereochemistry and geometry of the models and were found to be acceptable. Data collection and refinement statistics are presented in Table 1.

### Heme-binding analysis

Apo forms of wild-type or mutant recombinant IsdX1 (5 µM) were incubated with hemin chloride (5 µM – Sigma) in PBS, pH 7.4 for 10 minutes at 25°C. Reactions were then subjected to a wavelength scan from 250–560 nm using a DU800 spectrophotometer (Beckman-Coulter, London, UK) [29]. The relative amount of bound heme was calculated as: (T_399 nm_−C_399 nm_)/T_280 nm_, where T_399 nm_ = the absorbance maximum at 399 nm for samples containing IsdX1 with heme, C_399 nm_ = the absorbance maximum at 399 nm for samples containing heme only, and T_280 nm_ = the absorbance maximum at 280 nm for samples containing IsdX1 with heme. To measure the rates of heme dissociation, wild-type or mutant IsdX1 were re-constituted with heme, excess heme removed by gel filtration chromatography, and holo proteins (1 µM) mixed with apo-Mb (26 µM) at 25°C in PBS, pH 7.4. Dissociation rates are determined by measuring the increase in absorbance at 419 nm (apo-Mb) versus a reference, control wavelength (380 nm).

### Hemoglobin-binding analysis

The interaction of wild-type and mutant IsdX1 proteins with hemoglobin was measured using a BIAcore 3000 biosensor (Amersham Biosciences, Piscataway, NJ) [30]. Briefly, 100 µL of holo- or apo-hemoglobin (Sigma-H2500, 10 µM in 50 mM Tris-HCl, pH 7.0) was covalently coupled to a CM5 sensor chip at 25°C to a density of 4000 response units (RUs) using amine chemistry as previously described [85], [86]. Wild-type or mutant IsdX1 proteins (100, 200, 300, 350, or 500 nM) in HBS-N (0.01 M HEPES, 0.15 M NaCl, pH 7.4) were injected at 20 µL/min for 300 s at 25°C and response curves followed for a total of 800 seconds. A parallel injection of IsdX1 over a blank CM5 surface (no hemoglobin) was used to control for non-specific binding. Injections at each concentration were performed in triplicate, and the data from the 300 nM injection used to calculate the dissociation constants, which were determined using BIAevaluation 4.1 software (Amersham Biosciences) after fitting the data to a 1∶1 Langmuir binding model with d*R*/d*t* = *k*_a_ *C*(*R*_max_−*R*)−*k*_d_ *R*, where *R* is the SPR signal (in response units), *k*_a_ is the association rate constant (in M^−1^ s^−1^), *k*_d_ is the dissociation rate constant (in s^−1^), *C* is the concentration of holo-IsdX1 (in M), *R*_max_ is the maximum holo-IsdX1 binding capacity (in response units), and d*R*/d*t* is the rate of change of the SPR signal [87].

### B. anthracis growth experiments

Purified IsdX1 (wild-type or mutant proteins) were treated with HCl and methyl ethyl ketone to remove any endogenous heme [88]. Preparations were then dialyzed against 2-L of PBS (pH 7.4) and treated with 100 mg/mL Chelex-100 (Sigma) to remove any contaminating free iron. *B. anthracis* Sterne strain (34F2) harboring deletions in both *isdX1* and *isdX2* [26] were subcultured into 3-mL of LB plus kanamycin at 30°C. After 12-hours, cells were washed 2× with PBS and 5-µL of washed, normalized cells inoculated into 500-µL of RPMI (iron chelated by treatment with 100 mg/mL Chelex-100 for 12 hours) with or without hemoglobin (10 µM) in a 48-well Costar tissue-culture plate. Wild-type or mutant proteins (1 µM) were next added to each well and OD_600_ recorded at 2, 4, 6, and 8 hours at 37°C using a Tecan 200 Pro microplater reader. The results represent the mean and standard deviation of three independent experiments.

### Circular Dichroism Spectroscopy

Circular dicroism spectra of apo forms of wild-type and mutant IsdX1 were obtained using a JASCO-815 CD spectropolarimeter at 25°C [89], [90]. Protein samples were resuspended in PBS buffer, pH 7.4 at a concentration of approximately 50 µM. Far-UV spectra were recorded from 200–260 nm using a 1-mm path length at a scanning speed of 50 nm/min, with a bandwidth of 2 nm. Raw spectra are shown and represent the average accumulation of six scans.

### Accession numbers

The NCBI accession number for *isdX1* is NC_005945.1 (*B. anthracis* Sterne gene ID:2851614).

## Supporting Information

Figure S1 **Comparison of surface charge distribution of IsdX1 to the** ***S. aureus*** **(Sa) NEAT domain structures.** Molecular surface representation with electrostatic potential as shown from −70 eV (negative, red) to +70 eV (positive, blue). Heme is represented by stick model and Fe as an orange sphere, with carbon, oxygen and nitrogen atoms colored in purple, red and blue, respectively. The PDB codes are Ba-IsdX1 (3SIK), Sa-IsdH-N3 (2Z6F), Sa-IsdC (2O6P), Sa-IsdA (2ITF) and Sa-IsdB-N2 (3RTL).
(TIF)

Figure S2 **Comparison of NEAT domain structures.** Upper panel, Comparison of the heme-binding pocket of the IsdX1 NEAT domain to those of *S. aureus* (Sa) NEAT proteins. Heme and residues in close proximity are represented by stick model with heme carbon atoms in purple and Fe as an orange sphere (all oxygen, nitrogen, and sulfur atoms colored red, blue, and yellow, respectively). Lower panel, An alternative conformation of Arg-54 coordinated to heme-iron is shown.
(TIF)

Figure S3 **Spectral properties of wild-type and mutant IsdX1.** Wild-type (black) or S52A, S53A, R54A, or M55A (grey) IsdX1 were purified from *E. coli* and the absorbance properties from 250–650 nm analyzed immediately after purification.
(TIF)

Figure S4 **Far-UV CD analysis of wild-type and mutant IsdX1.** Spectra of apo forms of wild-type and mutant IsdX1 (50 µM) were obtained using a JASCO-815 CD spectropolarimeter at 25°C.[89], [90] Raw spectra are shown and represent the average accumulation of six scans.
(TIF)

Figure S5 **Comparison of crystal packing of apo-IsdX1 and holo-IsdX1.** (A) Crystal packing of apo-IsdX1. Ribbon representation with two molecules in the asymmetry unit (blue box), where β-strands and helices are colored pink and cyan, respectively. The black line represents the non-crystallographic symmetry between the two molecules where one observed protein-interactions. Symmetry molecules are colored in light pink. Tyr166 and Tyr170 in the heme-binding site are in stick representation with carbon, nitrogen and oxygen atoms colored white, blue and red respectively. The protein interface formed by crystallographic symmetry occurs at the heme-binding site of two molecules. (B) Crystal packing of holo-IsdX1. Ribbon representation with two molecules in the asymmetry unit (blue box), where β-strands and helices are colored yellow and red, respectively. The black line represents the non-crystallographic symmetry between the two molecules, where in contrast to apo-IsdX1, one observes no protein interface. Symmetry molecules are colored in light yellow. Tyr166 and Tyr170 in the heme-binding site are in stick representation with carbon, nitrogen and oxygen atoms colored white, blue and red respectively. Heme is in stick representation with carbon atoms in grey. The protein interface formed by crystallographic symmetry occurs between two heme molecules from crystallographic related molecules.
(TIF)
