# Correction: Differential Function of Lip Residues in the Mechanism and Biology of an Anthrax Hemophore

**DOI:** 10.1371/journal.ppat.1004122

**Published:** 2014-04-15

**Authors:** The PLOS Pathogens Staff

The eighth author’s name is spelled incorrectly. The correct name is Erol Bakkalbasi.
