# Supplementary figures and images for: Differential Function of Lip Residues in the Mechanism and Biology of an Anthrax Hemophore
Source: PLoS Pathog. 2012 Mar 8;8(3):e1002559. doi: 10.1371/journal.ppat.1002559 (PMC3297588; doi:10.1371/journal.ppat.1002559)

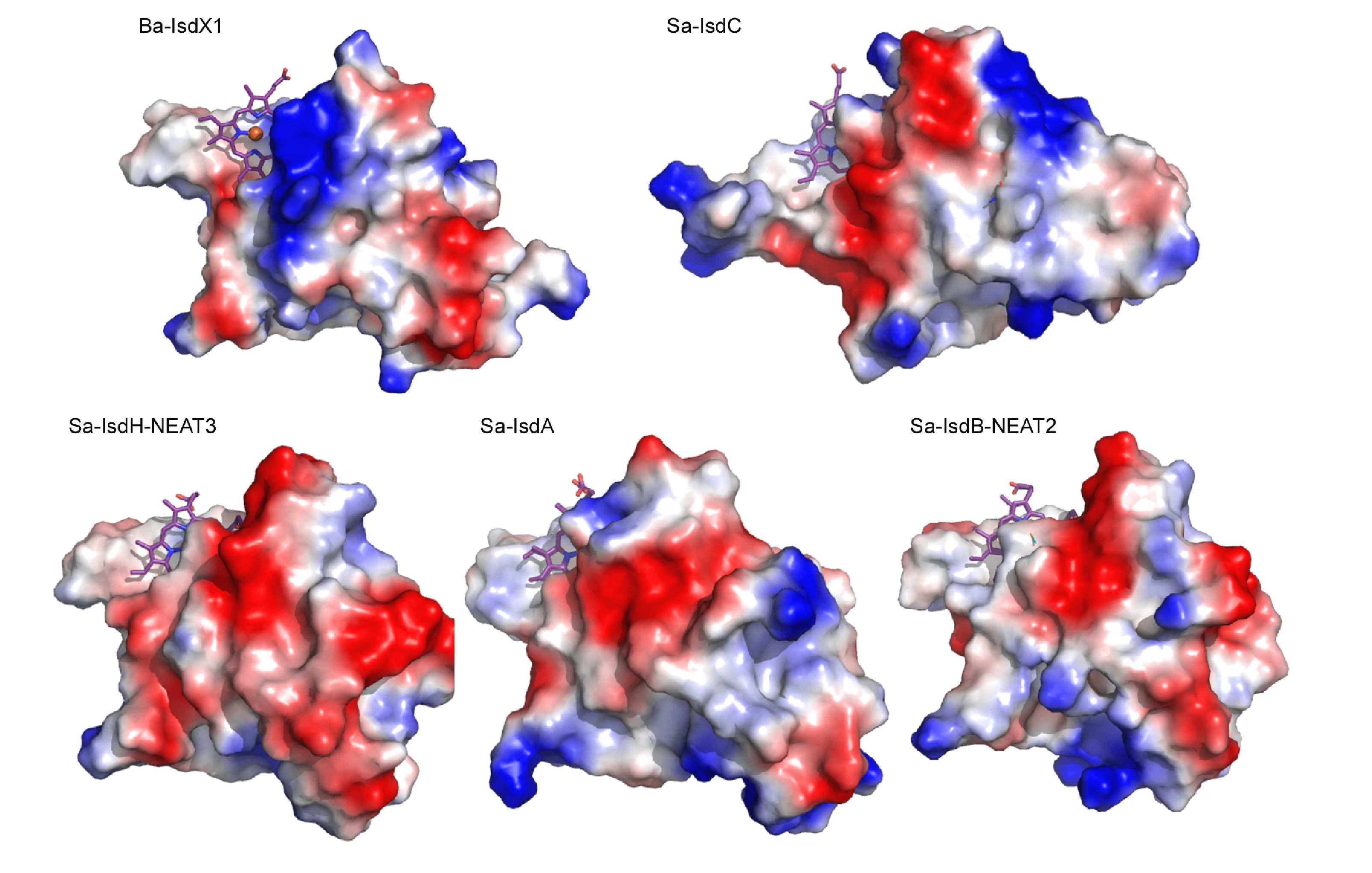

Supplement: Figure S1 — Comparison of surface charge distribution of IsdX1 to the S. aureus (Sa) NEAT domain structures. Molecular surface representation with electrostatic potential as shown from −70 eV (negative, red) to +70 eV (positive, blue). Heme is represented by stick model and Fe as an orange sphere, with carbon, oxygen and nitrogen atoms colored in purple, red and blue, respectively. The PDB codes are Ba-IsdX1 (3SIK), Sa-IsdH-N3 (2Z6F), Sa-IsdC (2O6P), Sa-IsdA (2ITF) and Sa-IsdB-N2 (3RTL). (TIF) [file ppat.1002559.s001.tif]

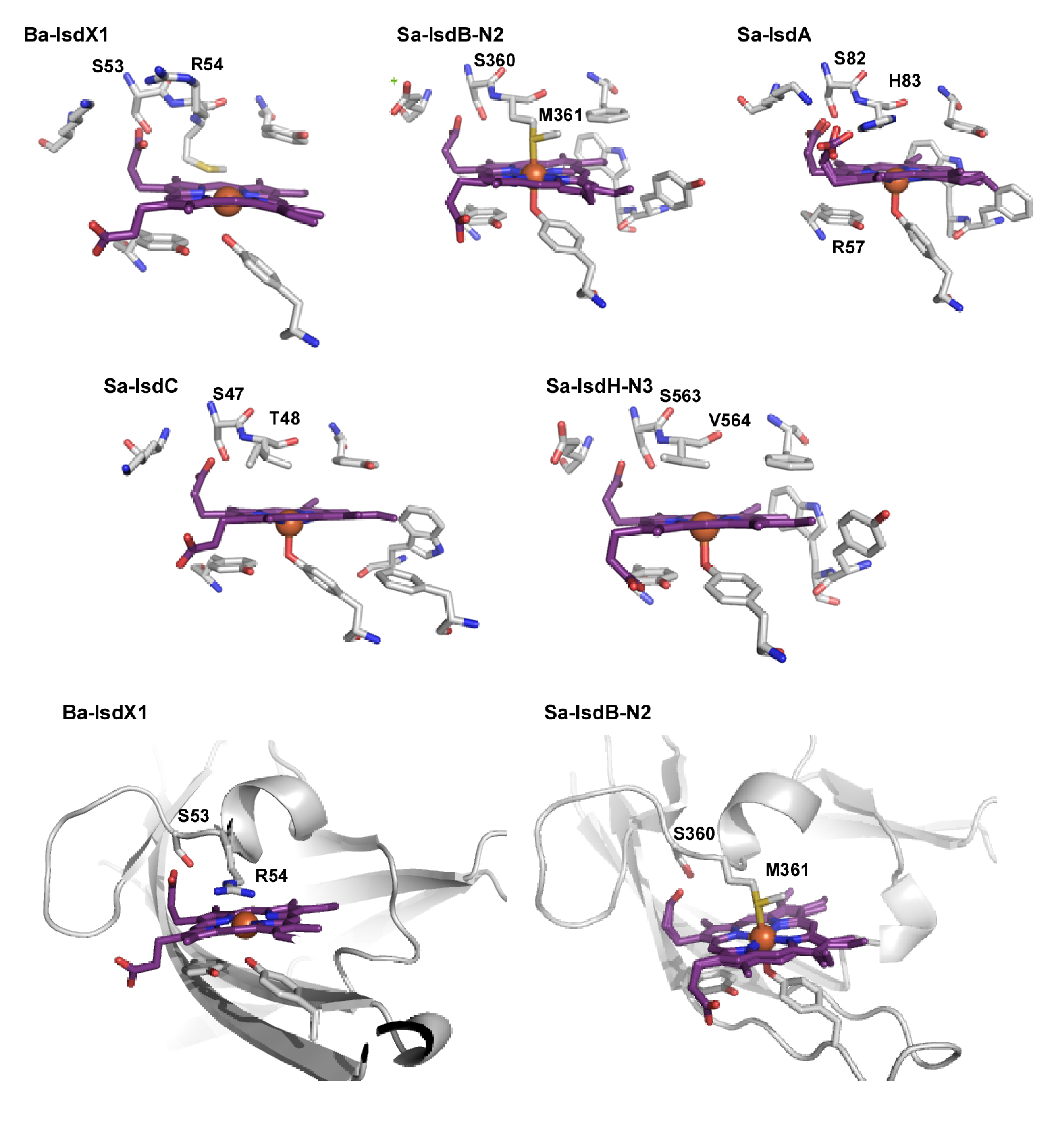

Supplement: Figure S2 — Comparison of NEAT domain structures. Upper panel, Comparison of the heme-binding pocket of the IsdX1 NEAT domain to those of S. aureus (Sa) NEAT proteins. Heme and residues in close proximity are represented by stick model with heme carbon atoms in purple and Fe as an orange sphere (all oxygen, nitrogen, and sulfur atoms colored red, blue, and yellow, respectively). Lower panel, An alternative conformation of Arg-54 coordinated to heme-iron is shown. (TIF) [file ppat.1002559.s002.tif]

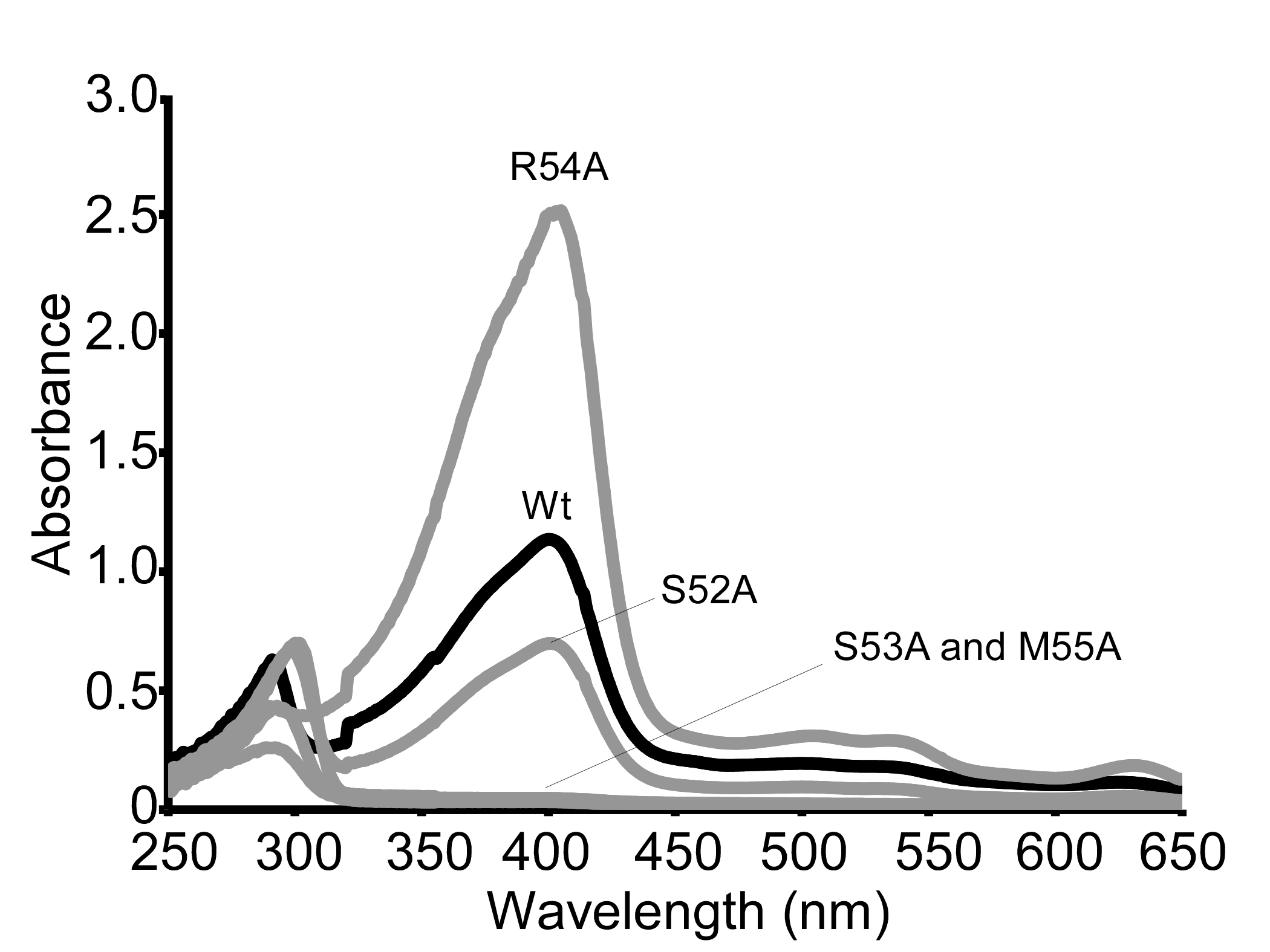

Supplement: Figure S3 — Spectral properties of wild-type and mutant IsdX1. Wild-type (black) or S52A, S53A, R54A, or M55A (grey) IsdX1 were purified from E. coli and the absorbance properties from 250–650 nm analyzed immediately after purification. (TIF) [file ppat.1002559.s003.tif]

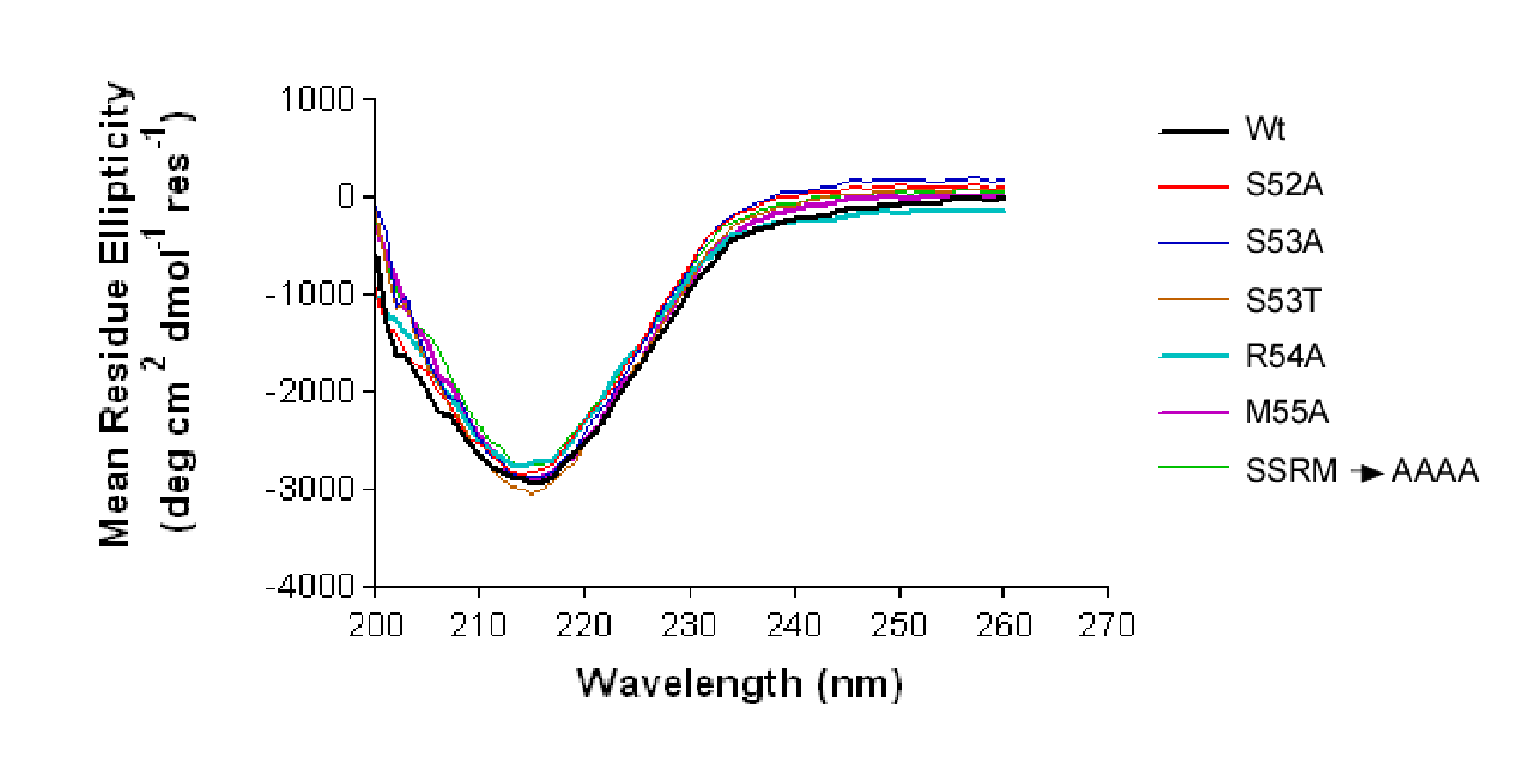

Supplement: Figure S4 — Far-UV CD analysis of wild-type and mutant IsdX1. Spectra of apo forms of wild-type and mutant IsdX1 (50 µM) were obtained using a JASCO-815 CD spectropolarimeter at 25°C.[89], [90] Raw spectra are shown and represent the average accumulation of six scans. (TIF) [file ppat.1002559.s004.tif]

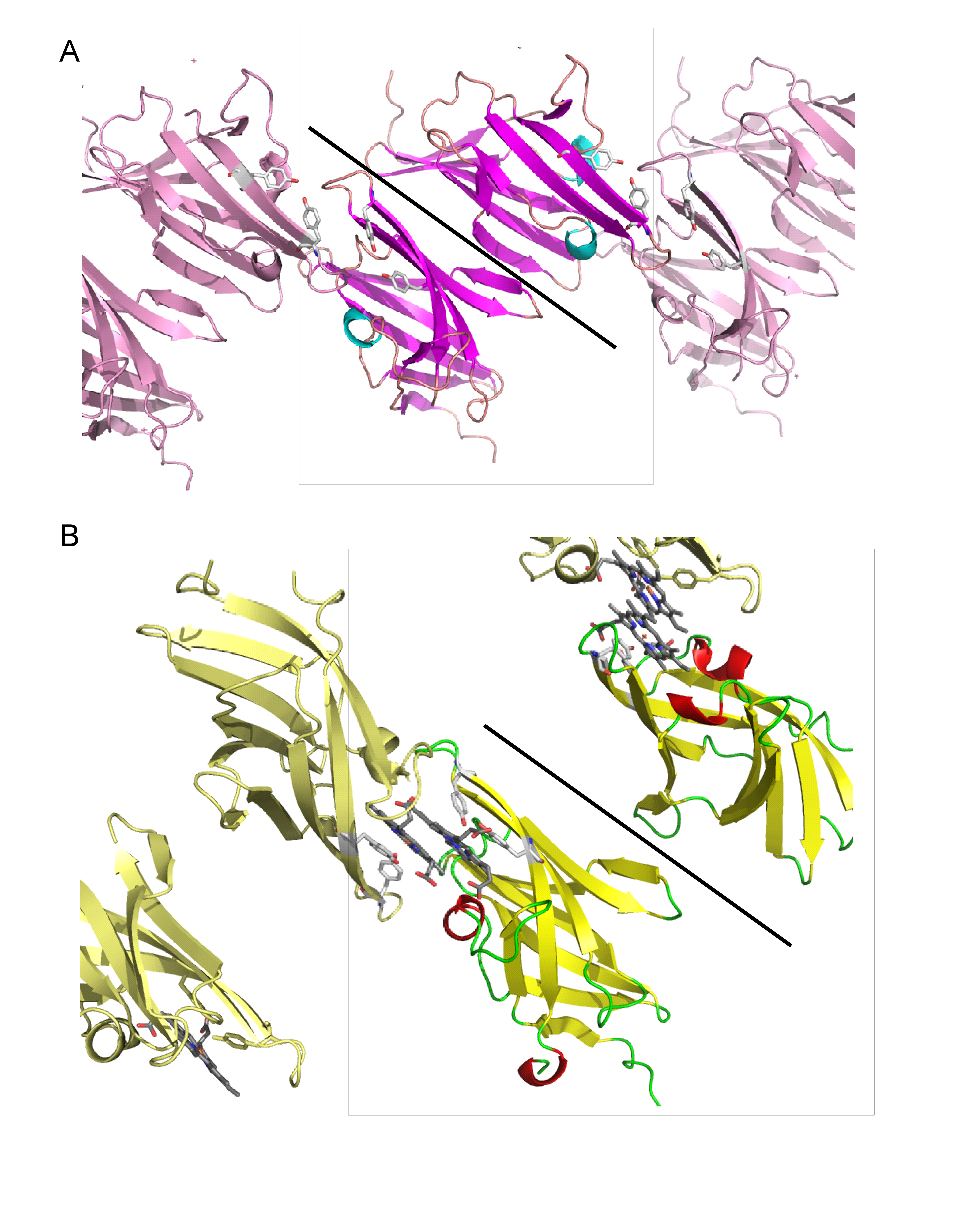

Supplement: Figure S5 — Comparison of crystal packing of apo-IsdX1 and holo-IsdX1. (A) Crystal packing of apo-IsdX1. Ribbon representation with two molecules in the asymmetry unit (blue box), where β-strands and helices are colored pink and cyan, respectively. The black line represents the non-crystallographic symmetry between the two molecules where one observed protein-interactions. Symmetry molecules are colored in light pink. Tyr166 and Tyr170 in the heme-binding site are in stick representation with carbon, nitrogen and oxygen atoms colored white, blue and red respectively. The protein interface formed by crystallographic symmetry occurs at the heme-binding site of two molecules. (B) Crystal packing of holo-IsdX1. Ribbon representation with two molecules in the asymmetry unit (blue box), where β-strands and helices are colored yellow and red, respectively. The black line represents the non-crystallographic symmetry between the two molecules, where in contrast to apo-IsdX1, one observes no protein interface. Symmetry molecules are colored in light yellow. Tyr166 and Tyr170 in the heme-binding site are in stick representation with carbon, nitrogen and oxygen atoms colored white, blue and red respectively. Heme is in stick representation with carbon atoms in grey. The protein interface formed by crystallographic symmetry occurs between two heme molecules from crystallographic related molecules. (TIF) [file ppat.1002559.s005.tif]
